# Maximum-Entropy Tools for Economic Fitness and Complexity

**DOI:** 10.3390/e20100743

**Published:** 2018-09-28

**Authors:** Ruben Krantz, Valerio Gemmetto, Diego Garlaschelli

**Affiliations:** 1 Lorentz Institute for Theoretical Physics, Leiden University, 2333 RA Leiden, The Netherlands; 2 IMT School for Advanced Studies, 55100 Lucca, Italy

**Keywords:** economic complexity, entropy, complex networks

## Abstract

The concepts of economic fitness and complexity, based on iterative and interdependent definitions of the quality of exporting countries and exported products, have led to novel insights into the dynamics of production and trade. A key step in the calculation of these quantities is the preliminary identification of statistically relevant country-product pairs.In this paper, we propose a method that could improve the current practice of filtering based on the revealed comparative advantage, by employing the maximum-entropy principle to construct an unbiased link weight probability distribution that, unlike the traditional thresholding method, allows for the statistical assessment of empirical trade volumes. The result is an adjusted geometric distribution for trade links that refines the revealed comparative advantage approach. This allows us to define the statistical significance of each trade link weight, leading to statistically supported trade link filtering decisions. Using this statistically justified filtering method, we have obtained results that are similar in nature to those that were found without this method, even though there are significant deviations in the details. In addition, the statistical information thus obtained on each trade link allows us to perform a spectral analysis of the export portfolio of individual economies.

## 1. Introduction

Economists have made many attempts at defining the competitiveness of economies on a national scale, starting with the “father of modern economy”, Adam Smith, in the wealth of nations. Approaches have ranged from attempting to point out the factors that make for a competitive economy, like Smith did, to an a posteriori analysis of the economic successfulness of a country, e.g., by measuring the gross domestic product (GDP). Thus far, none of these approaches have accomplished a comprehensive method of both explaining and measuring competitiveness. A recent attempt has been made by defining economic complexity, which explains why certain economies are more successful than others and gives a good estimate of the relative success they have.

### 1.1. Economic Complexity

Counter to standard economical theory, which states that while poorly developed countries specialize in exporting the least complex products, highly developed countries presumably produce solely the more complex products, the paper by Hidalgo and Hausmann [1] has shown that the latter have, instead, a highly diversified basket of export products, ranging from the most complex down to the simplest commodities. This called for a new approach, one that defines the competitiveness of a country’s economy on the diversity of the products it produces.

While acknowledging the pioneering work by Hidalgo and Hausmann [1], which in short states that a country’s “fitness” is simply determined by the sum of the “complexity” of its products and vice versa, it serves us best in our current effort to focus on the approach developed later by Tacchella et al. [2,3,4], which has proven more robust as it reaches a stable solution whereas the original algorithm does not.

The conceptual reasoning behind this method is that a commodity (product) that is produced only by fit economies can be labeled complex, while those that are produced by a large number of economies, with high and low fitness, are marked as less complex. Inversely, economies that export only simple commodities are taken to be the least fit, while those that export a diversified range of complex and non-complex commodities are labeled as fit. This allows a ranking of different countries along the line of their fitness. As product complexity relies on countries’ fitness and vice versa, this naturally is an iterative algorithm. Formally, it can be expressed as follows:

(1) $$\begin{array}{c} \left\{\begin{array}{c} {\tilde{F}}_{i}^{\left(n\right)}=\sum_{\alpha }M_{i}^{\alpha }Q^{\alpha (n-1)},\\ {\tilde{Q}}^{\alpha (n)}=\frac{1}{\sum_{i}M_{i}^{\alpha }\frac{1}{F_{i}^{\left(n-1\right)}}}, \end{array}\right. \end{array}$$

where both $F_{i}$ and $Q^{\alpha }$ start with a value of 1 for all countries and commodities at the first iteration. In this definition, *i* represents the exporting country, while α denotes the exported commodity. The iteration number is represented by *n*. A normalization step after each iteration ensures the divergence of both fitness and complexity:

(2) $$\begin{array}{c} \left\{\begin{array}{c} F_{i}^{\left(n\right)}=\frac{{\tilde{F}}_{i}^{\left(n\right)}}{{\langle {\tilde{F}}_{i}^{\left(n\right)}\rangle }_{i}},\\ Q_{\alpha }^{\left(n\right)}=\frac{{\tilde{Q}}^{\alpha (n)}}{{\langle {\tilde{Q}}^{\alpha (n)}\rangle }_{\alpha }}. \end{array}\right. \end{array}$$

It is important to note the role of the binary bipartite country-commodity matrix $M_{i}^{\alpha }$ in these defining equations. This matrix represents the bipartite network of countries and the commodities they do or do not export (represented by 1 or 0, respectively), which is derived from the world trade network (WTN). The WTN is a multi-layered network with a layer (regular network) for each commodity α, showing how much each exporting country *i* trades in that commodity with importing country *j*. This (weighted) WTN is represented by the weighted adjacency matrix *W* with components $w_{i,j}^{\alpha }$. In current practice, this matrix is first summed ($w_{i}^{\alpha }=\sum_{j}w_{i,j}^{\alpha }$) and then filtered to retrieve the matrix $M_{i}^{\alpha }$. In this paper, we will point out why the current approach is flawed at this point, and put forward an improved methodology to replace it.

### 1.2. Revealed Comparative Advantage: Current Practice and Flaws

In their criticism of the notions of fitness and complexity, Morrison et al. [5] point out the instability of the fitness and complexity algorithm. Their work shows that the addition or removal of a single product into the analysis can lead to significant changes in the resulting fitness of all countries, not only those who supposedly export it, leading them to question the usefulness of the algorithm. This emphasizes the importance of a well chosen filtering approach.

Thus far, the filtering of the weighted bipartite adjacency matrix to obtain its binary counterpart $M_{i}^{\alpha }$ has been limited to the straightforward application of the revealed comparative advantage (RCA), an economical concept first conceived by Balassa [6]. Conceptually, it is the share of a single country in the total trade of a certain commodity divided by the country’s share in the total world trade of all commodities. The mathematical definition of the RCA is rather straightforward:

(3) $$\begin{array}{c} {RCA}_{i}^{\alpha }=\frac{\frac{w_{i}^{\alpha }}{\sum_{i^{\prime }}w_{i^{\prime }}^{\alpha }}}{\frac{\sum_{\alpha^{\prime }}w_{i}^{\alpha^{\prime }}}{\sum_{i^{\prime }\alpha^{\prime }}w_{i^{\prime }}^{\alpha^{\prime }}}}. \end{array}$$

Originally, this measure determines the relative importance of the trade in a certain commodity for a country, as compared to other countries or other commodities that the country in scope trades. In the current application, it serves to determine whether a country is a relevant exporter of a commodity. For each country and commodity, when the RCA is larger then or equal to 1, the corresponding trade link in the country-commodity matrix has a value of 1 and 0 otherwise. This is formalized by the filtering rule:

(4) $$\begin{array}{c} M_{i}^{\alpha }=\left\{\begin{array}{c} 1,\;\mathrm{if}:\;{RCA}_{i}^{\alpha }\geq 1,\\ 0,\;\mathrm{if}:\;{RCA}_{i}^{\alpha }<1, \end{array}\right. \end{array}$$

which, with a slight change of perspective, can also be regarded as a comparison between the real world values $w_{i}^{\alpha }$ and the expected value yielded by an RCA based null model for the network

(5) $$\begin{array}{c} M_{i}^{\alpha }=\left\{\begin{array}{c} 1,\;\mathrm{if}:\;w_{i}^{\alpha }\geq \langle w_{i}^{\alpha }\rangle ,\\ 0,\;\mathrm{if}:\;w_{i}^{\alpha }<\langle w_{i}^{\alpha }\rangle , \end{array}\right. \end{array}$$

where the null model’s expectation values are created by equating ${RCA}_{i}^{\alpha }=1$:

(6) $$\begin{array}{c} \langle w_{i}^{\alpha }\rangle =\frac{\sum_{\alpha^{\prime }}w_{i}^{\alpha^{\prime }}\cdot \sum_{i^{\prime }}w_{i^{\prime }}^{\alpha }}{\sum_{i^{\prime }\alpha^{\prime }}w_{i^{\prime }}^{\alpha^{\prime }}}. \end{array}$$

The phrase null model in the context of graph theory encompasses a network that mimics the original network in some properties, but is randomized, or generalized, in all other. The null model is generally used for comparison to extract certain characteristics from the original network—in our current case, relevant trade links.

However, this RCA “null model” is chosen implicitly; moreover, in our opinion, this choice is not well-motivated and leads to flaws that can have implications all the way to the results of the complexity algorithm itself. Our critique is summarized by these three arguments:

- The RCA as a null model represents a fully connected or very dense network, as by the definition in Equation (6) it has a non-zero value for each *i* and α that have a non-zero total trade. In practice, this is the case for almost all links. In contrast, the world trade network is quite sparse with only 2–4% of all potential links realized throughout the analyzed years.
- The current definition of the RCA only applies to the bipartite network of countries and commodities, while the original world trade network contains another dimension of information, being the receiving importing country. Keeping in mind that a null model should mimic the original network, this importer dimension should also be represented in any appropriate null model—especially so, because the trade weight that the RCA null model would expect does not depend at all on the receiving country, while in reality this is of course of major importance (one would expect more trade to a country with a lot of incoming trade).
- Most importantly, the current methodology does not take into account the statistical significance of the filtered values. An RCA of over 1 could signify an important export product of a country, but could just as well be due to a statistical fluctuation through the years. This flaw is something that Tacchella et al. also partially realized (see supplement of [7]), leading them to develop a hidden Markov model approach to binarize the country-commodity matrix to reduce this noise. We choose a different path, keeping to the original data and performing a statistical analysis to keep noise at bay.

The innovation of this paper is that we replace the current method with a null model that extends the RCA to three dimensions (exporter, importer and commodity), mimics the original network in its sparsity by controlling the probability that a link exists, and includes a probability distribution (with the expected weight and variance thereof) for each link weight in order to make statistically justified filtering choices.

## 2. Methodology

Given the considerations in the introduction, we aim at stepping back from the country-commodity matrix to the underlying multiplex trade layers (a multiplex network is a network consisting of multiple layers with the same nodes but different links between these nodes in each layer). Hence, we will first extend the RCA to a layer-specific version dependent on the importer, as well as the exporter and the commodity, like in the original, in Section 2.1. Thereby, we introduce a multiplex null model, for which we will develop an unbiased weight distribution around the RCA expected value. Our path towards that goal heavily relies on the maximum likelihood method as described in [8,9]. As in other recent research in complex networks concerning economics and innovation [10,11], entropy plays an important role in this approach.

In an approach analogous to statistical mechanics, the idea of this method is to use Shannon–Gibbs entropy and the Lagrange multiplier technique to establish link probabilities. Given an ensemble of graphs that satisfy a set of topological constraints linked to the original graph, this approach allows us to establish the graph in the ensemble with the highest entropy, where the concept of maximum entropy in the network context means a graph with the least possible amount of information or graph-specific patterns [8]. The maximum likelihood method is applied to find the values for the Lagrange multipliers that ensure the constrained topological features are most likely to align with the real world graph. Using the Lagrange multipliers, we can define a link weight probability distribution for every link in the WTN, allowing a statistical filtering on the weights of links, providing an improved input for the country-commodity matrix used in the fitness and complexity algorithm.

However, the world trade network requires a specific approach as it is both a multiplex and a weighted network. Therefore, we will first develop a multiplex framework building on previous work in Section 2.2, before moving on to the core of our improvements in subsequent subsections.

### 2.1. The Extended RCA

Our criticism that the RCA only functions as a null model for the bipartite country-commodity network can be countered by a straightforward extension to the importer dimension. When one regards the denominator in Equation (6) as the normalization that ensures that $\sum_{i}\langle w_{i}^{\alpha }\rangle =\sum_{i}w_{i}^{\alpha }$ and $\sum_{\alpha }\langle w_{i}^{\alpha }\rangle =\sum_{\alpha }w_{i}^{\alpha }$, the obvious expression for the expectation value $\langle w_{i,j}^{\alpha }\rangle$ becomes

(7) $$\begin{array}{c} \langle w_{i,j}^{\alpha }\rangle =\frac{\sum_{j^{\prime },\alpha^{\prime }}w_{i,j^{\prime }}^{\alpha^{\prime }}\cdot \sum_{i^{\prime },\alpha^{\prime }}w_{i^{\prime },j}^{\alpha^{\prime }}\cdot \sum_{i^{\prime },j^{\prime }}w_{i^{\prime },j^{\prime }}^{\alpha }}{{\left(\sum_{i^{\prime },j^{\prime },\alpha^{\prime }}w_{i^{\prime },j^{\prime }}^{\alpha^{\prime }}\right)}^{2}}. \end{array}$$

This extended RCA can function as a null model for the weights of the world trade network in its full detail.

### 2.2. Extension to the Multiplex Network

The maximum likelihood method builds upon the concept of maximum entropy. This requires us to extend the graph’s Shannon–Gibbs entropy and with that, the graph probability, from a regular single layer network to a multiplex network. This was previously developed and described in [12]. We will express the probability that a multiplex network with the set of adjacency matrices $\left\{W^{\alpha }\right\}$ exists ($P(\left\{W^{\alpha }\right\})$) in terms of the single layer network probability $P^{\alpha }\left(W^{\alpha }\right)$, starting off by extending the Hamiltonian:

(8) $$\begin{array}{c} H(\left\{W^{\alpha }\right\})=\sum_{\alpha }H^{\alpha }\left(W^{\alpha }\right), \end{array}$$

(9) $$\begin{array}{c} S=-\sum_{\left\{W^{\alpha }\right\}}P\left(\left\{W^{\alpha }\right\}\right)\ln P\left(\left\{W^{\alpha }\right\}\right). \end{array}$$

Now, if the probability on a single layer in the multiplex is:

(10) $$\begin{array}{c} P^{\alpha }\left(W^{\alpha }\right)=\frac{e^{-H(\left\{W^{\alpha }\right\})}}{Z} \end{array}$$

(where *Z* is the partition function: $Z=\sum_{\left\{W^{\alpha }\right\}}e^{-H(\left\{W^{\alpha }\right\})}$). Then, the full multiplex graph probability is:

(11) $$\begin{array}{cc} P(\left\{W^{\alpha }\right\}) & =\prod_{\alpha }P^{\alpha }\left(W^{\alpha }\right)\\ & =\frac{\prod_{\alpha }e^{-H(\left\{W^{\alpha }\right\})}}{Z}. \end{array}$$

### 2.3. Link Weight Probability Distribution

With the formalities out of the way, we can move on to our goal of expressing a link weight probability distribution $q_{i,j}^{\alpha }\left(w_{i,j}^{\alpha }\right)$ that makes up the components of the (multiplex) graph probability

(12) $$\begin{array}{c} P(\left\{W^{\alpha }\right\})=\prod_{i,j,\alpha }q_{i,j}^{\alpha }\left(w_{i,j}^{\alpha }\right). \end{array}$$

Considering the fact that the weight of a trade link can theoretically range from zero to infinity, the parallel with Fermi statistics or a geometric probability distribution immediately comes to mind. We believe that this would be oversimplifying the practical situation, as it is hard to defend the viewpoint that the first traded weight has the same probability linked to it as all the following. We therefore follow [13] in their generalization of Bose and Fermi statistics, leading us to a modified geometric distribution that takes into account the difficulty of establishing the first trade link:

(13) $$\begin{array}{c} q_{i,j}^{\alpha }\left(w_{i,j}^{\alpha }\right)=\left\{\begin{array}{cc} p_{i,j}^{\alpha }{\left(r_{i,j}^{\alpha }\right)}^{w_{i,j}^{\alpha }-1}\cdot \left(1-r_{i,j}^{\alpha }\right), & \mathrm{if}:\;w_{i,j}^{\alpha }>0,\\ 1-p_{i,j}^{\alpha }, & \mathrm{if}:\;w_{i,j}^{\alpha }=0. \end{array}\right. \end{array}$$

In this distribution, $p_{i,j}^{\alpha }$ is the probability of establishing the link in the first place and $r_{i,j}^{\alpha }$ is the probability of adding a unit of weight.

This leaves us with the task of finding expressions for $p_{i,j}^{\alpha }$ and $r_{i,j}^{\alpha }$ in order to complete the link weight probability distribution (and be able to find the statistical significance of each actual link). We have developed three distinct approaches to finding a reasonable link existence probability ($p_{i,j}^{\alpha }$):

- Directed binary configuration model (DBCM),
- Multiplex directed binary configuration model (MDBCM),
- Strength-replaced MDBCM.

### 2.4. The Directed Binary Configuration Model

For all of these approaches, we follow the same basic rules of the maximum likelihood method described in [8], in order to arrive at the directed binary configuration model (DBCM) expression for $p_{i,j}^{\alpha }$ first. The rather straightforward extension to the MDBCM and the strength-replaced MDBCM will be discussed in the following subsections.

The maximum likelihood method is applied to construct an unbiased ensemble of all possible graphs {G} that resemble the original G* in a predefined topological property. As constructing a micro-canonical ensemble, where all the topological constraints are met exactly, can only be done numerically and not analytically, we opt for the computationally faster constructed canonical ensemble, where the expectation values of the topological constraints meet the real world originals. Thus, instead of the strict $\vec{C}\left(\left\{G\right\}\right)=\vec{C}\left(G*\right)$ (i.e., requiring that the topological properties $\vec{C}$ of all graphs in ensemble G are equal to those in the original graph), we require only that:

(14) $$\begin{array}{c} \langle \vec{C}\left(\left\{G\right\}\right)\rangle =\vec{C}\left(G*\right). \end{array}$$

In this subsection, we will develop the DBCM, which can be defined as the canonical ensemble constructed using the the in- and out-degree—the simplest first order topological property—of all nodes of only a single layer of the full network as constraint. This means that, in the current application of the WTN, the DBCM will be applied layer for layer. For our current purposes, we limit ourselves to a binary representation of a graph, constraining degrees instead of strengths. Thus, the sparsity of the original graph will be conserved:

(15) $$\begin{array}{c} \langle \vec{k}\left(\left\{G\right\}\right)\rangle =\vec{k}\left(G*\right) \end{array}$$

or more specifically, for directed graphs like the WTN:

(16) $$\begin{array}{c} \langle {\vec{k}}_{in/out}\left(\left\{G\right\}\right)\rangle ={\vec{k}}_{in/out}\left(G*\right), \end{array}$$

where $\vec{k}$ is the vector containing degrees $k_{i}$ of all nodes *i*.

To ensure a completely unbiased randomization of the canonical ensemble, we require that the probability P(G) that a graph **G** exists maximizes the Shannon–Gibbs entropy, subject to the constraint defined above (Equation (16)) and enforcing a normalized probability distribution:

(17) $$\begin{array}{cc} S & \equiv -\sum_{G}P\left(G\right)\ln P\left(G\right),\;\mathrm{where}:\\ \sum_{G}P\left(G\right) & =1. \end{array}$$

These requirements can be met by introducing into the graph probability expression a set of Lagrange multipliers $\vec{\theta }=\left\{\theta_{a}\right\}$ and $\vec{\phi }=\left\{\phi_{a}\right\}$ that enforce the ensemble constraints (Equation (16)). The general expression for the graph probability then becomes

(18) $$\begin{array}{c} P(G|\vec{\theta },\vec{\phi })=\frac{e^{-H(G|\vec{\theta },\vec{\phi })}}{Z(\vec{\theta },\vec{\phi })}, \end{array}$$

where the Hamiltonian is defined as the product of the Lagrange multipliers and the constraints

(19) $$\begin{array}{c} H(G|\vec{\theta },\vec{\phi })\equiv \sum_{i,j}\theta_{i}k_{i,out}\left(G\right)+\phi_{j}k_{j,in}\left(G\right)=\vec{\theta }\cdot {\vec{k}}_{out}\left(G\right)+\vec{\phi }\cdot {\vec{k}}_{in} \end{array}$$

and the partition function $Z(\vec{\theta })$ normalizes the probability distribution

(20) $$\begin{array}{c} Z(\vec{\theta },\vec{\phi })=\sum_{G}e^{-H(G|\vec{\theta },\vec{\phi })} \end{array}$$

and, finally, a topological property’s expectation value can be expressed using the graph probability

(21) $$\begin{array}{c} {\langle X\rangle }_{\vec{\theta },\vec{\phi }}\equiv \sum_{G}X\left(G\right)P\left(G|\vec{\theta },\vec{\phi }\right). \end{array}$$

The key step to fit the model that is defined by Equation (18) to the real world graph G* is to tune the Lagrange multipliers so that the likelihood of retrieving that original graph is maximized. Following [14], this is achieved by setting

(22) $$\begin{array}{cc} {\langle \vec{C}\left(\left\{G\right\}\right)\rangle }_{\vec{\theta *},\vec{\phi *}}=\sum_{G}\vec{C}\left(G\right)P\left(G|\vec{\theta },\vec{\phi }\right) & =\vec{C}\left(G*\right), \end{array}$$

which, in our configuration model approach, leads to fixing:

(23) $$\begin{array}{c} \left\{\begin{array}{c} \sum_{G}{\vec{k}}_{out}\left(G\right)P\left(G|\vec{\theta },\vec{\phi }\right)={\vec{k}}_{out}\left(G*\right),\\ \sum_{G}{\vec{k}}_{in}\left(G\right)P\left(G|\vec{\theta },\vec{\phi }\right)={\vec{k}}_{in}\left(G*\right). \end{array}\right. \end{array}$$

The key to establishing the Lagrange multipliers using the above expression is factorizing the graph probability into local components $P\left(G|\vec{\theta },\vec{\phi }\right)=\prod_{i,j}P_{ij}\left(g_{i,j}|\vec{\theta },\vec{\phi }\right)$, by rewriting the Hamiltonian:

(24) $$\begin{array}{cc} H(G|\vec{\theta },\vec{\phi }) & =\sum_{i,j}\theta_{i}k_{i,out}\left(G\right)+\phi_{j}k_{j,in}\left(G\right)\\ & =\sum_{i,j}\left(\theta_{i}+\phi_{j}\right)g_{i,j}, \end{array}$$

which leads to:

$$\begin{array}{cc} P(G|\vec{\theta },\vec{\phi }) & =\frac{e^{-\sum_{i,j}\left(\theta_{i}+\phi_{j}\right)g_{i,j}}}{\sum_{G}e^{-\sum_{i,j}\left(\theta_{i}+\phi_{j}\right)g_{i,j}}}\\ & =\frac{\prod_{i,j}{\left(e^{-\theta_{i}-\phi_{j}}\right)}^{g_{i,j}}}{\prod_{i,j}1+e^{-\theta_{i}-\phi_{j}}} \end{array}$$

(as $g_{i,j}$ is either 0 or 1 in a binary graph). Then, substituting the Lagrange multiplier exponentials with what we would call “hidden variables” $x_{i}=e^{-\theta_{i}}$ and $y_{j}=e^{-\phi_{j}}$

$$\begin{array}{c} =\prod_{i,j}\left(\frac{{\left(x_{i}y_{j}\right)}^{g_{i,j}}}{1+x_{i}y_{j}}\right)\\ =\prod_{i,j}\left(\frac{{\left(x_{i}y_{j}\right)}^{g_{i,j}}}{1+x_{i}y_{j}}\cdot {\left(\frac{1+x_{i}y_{j}}{1+x_{i}y_{j}}\right)}^{\left(g_{i,j}-1\right)}\right)\\ =\prod_{i,j}\left({\left(\frac{x_{i}y_{j}}{1+x_{i}y_{j}}\right)}^{g_{i,j}}\cdot {\left(1-\frac{x_{i}y_{j}}{1+x_{i}y_{j}}\right)}^{\left(1-g_{i,j}\right)}\right) \end{array}$$

(25) $$\begin{array}{c} =\prod_{i,j}p_{i,j}^{g_{i,j}}{\left(1-p_{i,j}\right)}^{\left(1-g_{i,j}\right)} \end{array}$$

(26) $$\begin{array}{c} =\prod_{i,j}P_{ij}\left(g_{i,j}|\vec{\theta },\vec{\phi }\right), \end{array}$$

where $p_{i,j}=\frac{x_{i}y_{j}}{1+x_{i}y_{j}}$ represents the probability that a link from *i* to *j* is present and therefore the success probability in the Bernoulli probability function $P_{ij}\left(g_{i,j}|\vec{\theta },\vec{\phi }\right)$.

We can now rewrite Equation (23) using the factorized version of $P(G|\vec{\theta },\vec{\phi })$ to get to expressions that we can use to numerically determine $x_{i}$ and $y_{j}$:

(27) $$\begin{array}{c} \langle g_{i,j}\rangle =\sum_{g}g_{i,j}p_{i,j}=p_{i,j}, \end{array}$$

leading to:

(28) $$\begin{array}{c} \left\{\begin{array}{c} \langle {\vec{k}}_{i,out}\left(G\right)\rangle =\sum_{j}p_{i,j}={\vec{k}}_{i,out}\left(G*\right),\\ \langle {\vec{k}}_{j,in}\left(G\right)\rangle =\sum_{i}p_{i,j}={\vec{k}}_{j,in}\left(G*\right). \end{array}\right. \end{array}$$

This is the mechanism behind the maximum likelihood method to build a statistically unbiased null model based on a single real world example, for a single layer, binary, directed network. However, the goal of this section was to find an expression for the link presence probability $p_{i,j}^{\alpha }$, for the link i,j in each layer α of the multiplex WTN, which was required in Equation (13). The key lies in simply solving the equivalent of Equation (28) generalized for the multiplex network numerically:

(29) $$\begin{array}{c} \left\{\begin{array}{c} \sum_{j}p_{i,j}^{\alpha }={\vec{k}}_{i,out}^{\alpha }\left(G*\right),\\ \sum_{i}p_{i,j}^{\alpha }={\vec{k}}_{j,in}^{\alpha }\left(G*\right), \end{array}\right.\;\mathrm{where}:\;p_{i,j}^{\alpha }=\frac{x_{i}^{\alpha }y_{j}^{\alpha }}{1+x_{i}^{\alpha }y_{j}^{\alpha }}. \end{array}$$

### 2.5. Multiplex Directed Binary Configuration Model

By construction, the DBCM is applied to each individual layer of the multiplex WTN separately. This implies that the null model that is thus created relies—as far as the expression for $p_{i,j}^{\alpha }$ goes—only on characteristics of that layer. This implies that only the trade activities of each country within a certain commodity are considered, while any general, inter-layer characteristics of a country in general are ignored. On top of that, solving the set of Equations (29) for all layers means finding 2n·l hidden variables (where n= number of nodes and l= number of layers), whereas this can be limited to 2n+l in the MDBCM.

With the goal of treating the WTN as a whole, instead of a large number of single layer networks, we can also construct a null model that takes cross-layer properties into account. This is achieved in the multiplex directed binary configuration model (MDBCM) by setting a whole new set of constraints to the configuration model, this time including the total amount of links in each layer (${\vec{L}}^{\alpha }$, referred to as the “layer degree”). Also note that the constrained out- and in-degrees are now replaced by their totals over all layers α:

(30) $$\begin{array}{c} \left\{\begin{array}{c} \langle {\vec{k}}_{i,out}^{tot}\left(G\right)\rangle ={\vec{k}}_{i,out}^{tot}\left(G*\right)=\sum_{j,\alpha }g_{i,j}^{\alpha },\\ \langle {\vec{k}}_{j,in}^{tot}\left(G\right)\rangle ={\vec{k}}_{j,in}^{tot}\left(G*\right)=\sum_{i,\alpha }g_{i,j}^{\alpha },\\ \langle {\vec{L}}^{\alpha }\left(G\right)\rangle ={\vec{L}}^{\alpha }\left(G*\right)=\sum_{i,j}g_{i,j}^{\alpha }. \end{array}\right. \end{array}$$

In general, we can follow the steps described in the previous section for the DBCM with just minor adjustments in the Hamiltonian, which will eventually lead to a new set of defining expressions for $p_{i,j}^{\alpha }$

(31) $$\begin{array}{cc} H(G|\vec{\theta },\vec{\phi },\vec{\zeta }) & =\sum_{i,j,\alpha }\theta_{i}k_{i,out}^{tot}\left(G\right)+\phi_{j}k_{j,in}^{tot}\left(G\right)+\zeta^{\alpha }L^{\alpha }\left(G\right) \end{array}$$

(32) $$\begin{array}{c} =\sum_{i,j,\alpha }\left(\theta_{i}+\phi_{j}+\zeta^{\alpha }\right)g_{i,j}^{\alpha }. \end{array}$$

Following the same steps as in the above derivation for the DBCM and substituting the Lagrange multipliers with the hidden variables $x_{i}=e^{-\theta_{i}}$, $y_{j}=e^{-\phi_{j}}$ and $z^{\alpha }=e^{-\zeta^{\alpha }}$ as before, we find:

(33) $$\begin{array}{c} p_{i,j}^{\alpha }=\frac{x_{i}y_{j}z^{\alpha }}{1+x_{i}y_{j}z^{\alpha }}. \end{array}$$

The construction of the MDBCM is then limited to the simple operation of numerically solving the following set of defining equations, in parallel to Equation (29):

(34) $$\begin{array}{c} \left\{\begin{array}{c} \sum_{j,\alpha }p_{i,j}^{\alpha }={\vec{k}}_{i,out}^{tot}\left(G*\right),\\ \sum_{i,\alpha }p_{i,j}^{\alpha }={\vec{k}}_{j,in}^{tot}\left(G*\right),\\ \sum_{i,j}p_{i,j}^{\alpha }={\vec{L}}^{\alpha }\left(G*\right), \end{array}\right. \end{array}$$

which clearly indicates the dependence of $p_{i,j}^{\alpha }$ on the total out- and in-degrees as well as the layer degrees. The former two, as required at the outset of the MDBCM, represent the network wide characteristics of individual nodes, while the latter represents inter-layer variations.

### 2.6. Strength-Replaced MDBCM

The final implementation of the maximum likelihood method that we have developed builds upon the MDBCM, instead of starting of with a new set of constraints. As the name suggests, we will replace two of the hidden variables by strengths. This is common practice in the use of the configuration model, where hidden variables are often replaced by some sort of “fitness” of the nodes. (Note that in this sense fitness means a measure of the performance of a node from a network theory point of view. This use of the term fitness is inherited from previous work [15,16]).

In our case, we will replace the Lagrange multiplier $x_{i}$ by the total out-strength of the corresponding node *i*, and $y_{j}$ by the total in-strength of node *j*

(35) $$\begin{array}{c} x_{i}\to s_{i,out}^{tot}\left(G*\right)\equiv \sum_{j,\alpha }w_{i,j}^{\alpha }\left(G*\right), \end{array}$$

(36) $$\begin{array}{c} y_{j}\to s_{j,in}^{tot}\left(G*\right)\equiv \sum_{i,\alpha }w_{i,j}^{\alpha }\left(G*\right), \end{array}$$

(37) $$\begin{array}{c} p_{i,j}^{\alpha }=\frac{s_{i,out}^{tot}s_{j,in}^{tot}z^{\alpha }}{1+s_{i,out}^{tot}s_{j,in}^{tot}z^{\alpha }}. \end{array}$$

Besides this replacement, there are no changes with respect to the previously discussed MDBCM. An advantage of this method is that it would be easier for laymen to understand and that it is simpler and theoretically faster to solve numerically, with only one set of hidden variables. For the scope of this paper, we will focus on the more rigorous MDBCM.

### 2.7. The Weight Unit Probability

So far, we have covered one out of two missing components of the geometric link weight probability distribution (Equation (13)), while $r_{i,j}^{\alpha }$ remains to be defined. We will exploit the choice of the extended RCA as the weight expectation value (Equation (7)) to solve Equation (13) for $r_{i,j}^{\alpha }$, while keeping $p_{i,j}^{\alpha }$ generic as we have several possible definitions for it:

(38) $$\begin{array}{cc} \langle w_{i,j}^{\alpha }\rangle & =\sum_{w\geq 0}q_{i,j}^{\alpha }\left(w_{i,j}^{\alpha }\right)\cdot w_{i,j}^{\alpha }\\ & =p_{i,j}^{\alpha }\cdot \sum_{w\geq 0}{\left(r_{i,j}^{\alpha }\right)}^{w_{i,j}^{\alpha }-1}\cdot \left(1-r_{i,j}^{\alpha }\right)\cdot w_{i,j}^{\alpha }\\ & =\frac{p_{i,j}^{\alpha }\cdot \left(1-r_{i,j}^{\alpha }\right)}{r_{i,j}^{\alpha }}\sum_{w>0}{\left(r_{i,j}^{\alpha }\right)}^{w_{i,j}^{\alpha }}\cdot w_{i,j}^{\alpha }\\ & =\frac{p_{i,j}^{\alpha }\cdot \left(1-r_{i,j}^{\alpha }\right)}{r_{i,j}^{\alpha }}\cdot \frac{r_{i,j}^{\alpha }}{{\left(r_{i,j}^{\alpha }-1\right)}^{2}}\\ & =\frac{p_{i,j}^{\alpha }}{1-r_{i,j}^{\alpha }} \end{array}$$

and therefore:

(39) $$\begin{array}{c} r_{i,j}^{\alpha }=1-\frac{p_{i,j}^{\alpha }}{\langle w_{i,j}^{\alpha }\rangle }. \end{array}$$

### 2.8. Statistical Significance

Now, we can return to our initial goal of replacing the crude RCA filtering method with one that gives a statistical justification. To that aim, we need the statistical significance of each real world link, as compared with the link weight probability distribution derived above. We will express this in *z*-scores: the number of standard deviations σ a weight deviates from the expected weight. Instead of filtering on RCA≥1, we can from then on apply the filter z≥τ, where τ is a chosen threshold. The values of the country-commodity matrix remain 1 for successful filtering and 0, otherwise, as was the case with the RCA.

As we intend to replace the bipartite country-commodity RCA as a filter applied before the fitness and complexity algorithm, we will limit ourselves to the bipartite *z*-score instead of finding the full three-dimensional version—which is trivial after the following derivation of the bipartite one:

(40) $$\begin{array}{cc} z\left(w_{i}^{\alpha }\right) & =\frac{w_{i}^{\alpha }-\langle w_{i}^{\alpha }\rangle }{\sigma \left(w_{i}^{\alpha }\right)}\\ & =\frac{\sum_{j}w_{i,j}^{\alpha }-\sum_{j}\langle w_{i,j}^{\alpha }\rangle }{\sqrt{\sum_{j}\sigma^{2}\left(w_{i,j}^{\alpha }\right)}}, \end{array}$$

requiring us to find an expression for the variance:

(41) $$\begin{array}{c} \sigma^{2}\left(w_{i,j}^{\alpha }\right)=\langle {\left(w_{i,j}^{\alpha }\right)}^{2}\rangle -{\langle w_{i,j}^{\alpha }\rangle }^{2}, \end{array}$$

(42) $$\begin{array}{cc} \langle {\left(w_{i,j}^{\alpha }\right)}^{2}\rangle & =\sum_{w\geq 0}q_{i,j}^{\alpha }\left(w_{i,j}^{\alpha }\right)\cdot {\left(w_{i,j}^{\alpha }\right)}^{2},\\ & =\frac{p_{i,j}^{\alpha }\cdot \left(1-r_{i,j}^{\alpha }\right)}{r_{i,j}^{\alpha }}\sum_{w>0}{\left(r_{i,j}^{\alpha }\right)}^{w_{i,j}^{\alpha }}\cdot {\left(w_{i,j}^{\alpha }\right)}^{2}\\ & =\frac{p_{i,j}^{\alpha }\cdot \left(1-r_{i,j}^{\alpha }\right)}{r_{i,j}^{\alpha }}\cdot -\frac{r_{i,j}^{\alpha }\left(r_{i,j}^{\alpha }-1\right)}{{\left(r_{i,j}^{\alpha }-1\right)}^{3}}\\ & =\langle w_{i,j}^{\alpha }\rangle \cdot \frac{1+r_{i,j}^{\alpha }}{1-r_{i,j}^{\alpha }}\\ & =\frac{2{\langle w_{i,j}^{\alpha }\rangle }^{2}}{p_{i,j}^{\alpha }}-\langle w_{i,j}^{\alpha }\rangle , \end{array}$$

thus:

(43) $$\begin{array}{cc} \sigma^{2}\left(w_{i,j}^{\alpha }\right) & =\frac{2-p_{i,j}^{\alpha }}{p_{i,j}^{\alpha }}{\langle w_{i,j}^{\alpha }\rangle }^{2}-\langle w_{i,j}^{\alpha }\rangle . \end{array}$$

### 2.9. Practical Implementation

Now that we have formally derived all the necessary components of the null model: the extended version of the RCA, the link weight probability distribution and the expression for the *z*-score that follows from it, we will briefly describe the practical implementation of the complete method in a comprehensive list:

1. Calculate $\langle w_{i,j}^{\alpha }\rangle$ for each link using the extended RCA as defined in Equation (7).
2. Find the hidden variables—and with that, $p_{i,j}^{\alpha }$—applying either the DBCM or the (regular or strength-replaced) MDBCM, by solving Equations (29) or (34), respectively.
3. Combine $\langle w_{i,j}^{\alpha }\rangle$ and $p_{i,j}^{\alpha }$ in Equation (43) to find the variance on each link i,j,α.
4. Use Equation (40) to find the *z*-score of each country-commodity pair i,α and filter all links in the bipartite network using a threshold on the *z*-score (typically z≥1, z≥2 or similar).
5. Apply the fitness and complexity algorithm as developed Tacchella et al. in [2].

## 3. Results

In this section, we will show that even though we propose a change to current practice that requires theoretical and methodological adjustments, there are no fundamental implications for the results. However, while they remain similar in nature, there are obvious discrepancies between the results of the original and our statistically justified approach—which tells us that it was, indeed, necessary to develop this more rigorous method. Furthermore, we will show a new set of results that are a direct outcome of our new approach, which can tell us more about the performance of each individual economy on a detailed level. Note that more details about the datasets used in this research are listed in Section 5. An important detail to mention here is that we have opted to apply the MDBCM method for finding $p_{i,j}^{\alpha }$, as this has proven to be faster than the DBCM and takes cross-layer patterns into account. We have chosen it over its strength replaced counterpart for the present, as it is theoretically the most profound of the two, leaving a thorough comparison for later concern.

### 3.1. Comparison with Previous Results

We have two goals in this comparison with the results that were achieved in [2,3,4] among others: firstly, we will shortly show that we have been able to reproduce their results (by applying a z≥0 filter, which is equal to the original RCA filtering method) and secondly we will show that, with the introduction of larger *z*-score threshold, the complexity algorithm still yields results of the same nature—while, naturally, we will also highlight the discrepancies.

#### 3.1.1. Evolution of Fitness and Complexity

**Figure 1:**
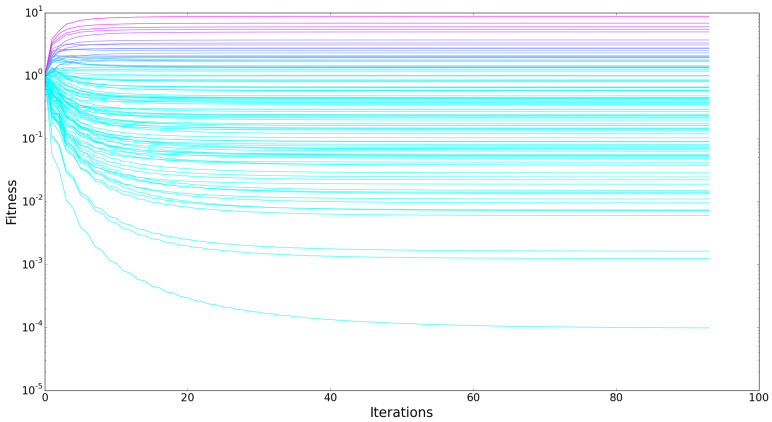
Evolution of country fitnesses throughout the iterations of the fitness and complexity algorithm both clearly show a convergence to a fixed point (using z≥0 for exact replication of revealed comparative advantage (RCA) filtering).

An indication of the improved working of the algorithm proposed by Tacchella et al. [2,3,4] is the evolution of the fitness and complexity indicators throughout the iterations of the algorithm. The graph in Figure 1 shows a clear divergence of fitness in the earlier iterations, while it converges to a fixed point for all countries in the end. Similar behavior is observed for complexity. This indicates the correct reproduction of their algorithm.

#### 3.1.2. Ranking Countries by Fitness

One of the main results presented in [2,3,4] is the ranking of countries according to their fitness. Note that the absolute fitness is of no real interest due to the normalization in each iteration of the algorithm. We have compared a top 10 ranking as found earlier using data from 2010 and the ranking we have produced using the 2012 data, as shown in the table below (Table 1). The discrepancies can largely be attributed to two differences: obviously the different years in scope, and the higher granularity of the data we have used. This table shows that, even though discrepancies are expected, the results are rather similar in nature.

#### 3.1.3. Correlation with GDP per Capita

**Figure 2:**
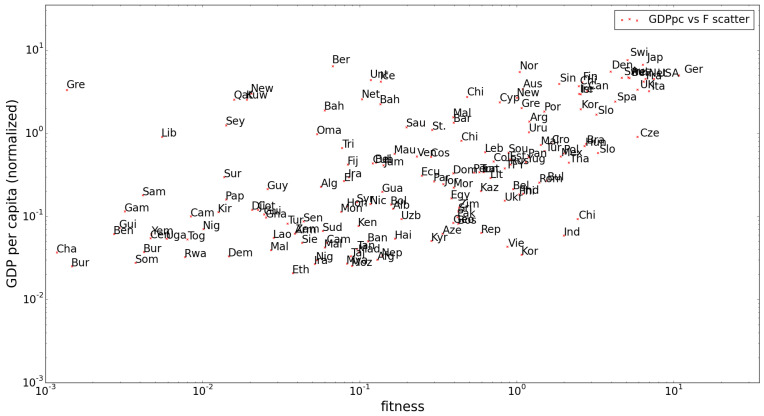
The normalized gross domestic product (GDP) per capita and fitness in 1995 are correlated with a correlation coefficient of 0.64 in the reproduction of the original (RCA filtered) results, with a standard error of 0.052 (which is substantial on this scale).

To conclude the comparison with the original results, we show that we have reproduced the global correlation that was found between the fitness of each country and its GDP per capita in Figure 2. We will not go into the supposed significance of this correlation, which in the first place was disputed in [5] and could be further questioned after a comparison with similar plots using higher *z*-score filters, as we will show in the following section.

### 3.2. Results with Filtering on Higher Statistical Significance

**Figure 3:**
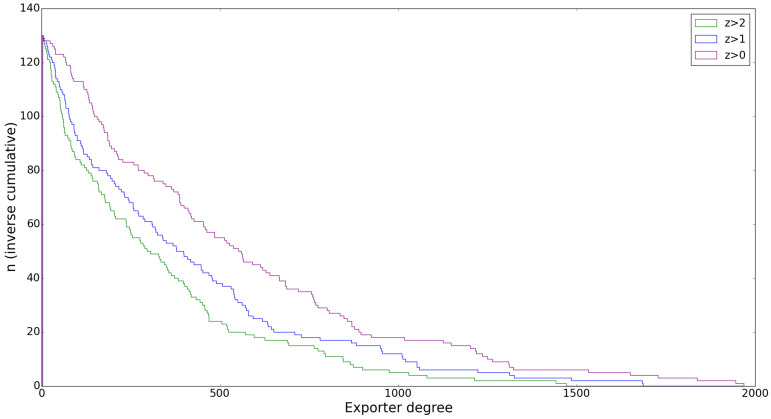
The inverse cumulative degree distribution of exporting countries in 2012 after filtering with different *z*-score thresholds clearly exposes the increased sparseness of the network after filtering.

We will cover the new results we obtained using a higher statistical significance threshold in the filtering procedure (z≥a where *a* is an integer larger than 0) in this subsection, emphasizing that although some discrepancies between the original results and these statistically justified ones do occur, they remain similar in nature. We would like to point out some practical implications of a stricter filtering procedure first, as these will help to clarify any different results. Naturally, when we take a higher filtering threshold, say z≥1 instead of z≥0, more of the links of the original network will be left out of the resulting matrix $M_{i}^{\alpha }$, which will become even sparser. This is can be seen in Figure 3, showing that there is a sharper drop in the out-degrees of the exporters as a smaller fraction of the commodities they export are passed through the filter.

The impact of the increased threshold is more practically illustrated by looking into the products that are allowed by one threshold value, but denied by another. For example, in 2004, the Netherlands had a *z*-score greater than zero (i.e., RCA filtering) for the products in Table 2 (and many others), but these did not pass a z≥1 filter. Some of these products would have been an important part of Dutch export several decades ago, but are clearly no longer as relevant (e.g., fish and electric lamps). Others are harder to interpret.

We will see that the impact of this sparsifying of $M_{i}^{\alpha }$ varies per country. Based on trivial statistics, one could expect that eliminating a certain fraction of all links for countries with a small original amount of trade links would lead to more extreme variations than eliminating that same fraction from countries highly connected countries. This intuition proves right, as we see that, for different *z*-score thresholds, the resulting top 10 countries in fitness ranking is relatively stable, while larger variations occur while going down the ranking. This is clearly shown in Table 3. Countries that perform steadily while we raise the *z*-score threshold thereby prove to have a more robust economy. This “robustness” can be visualized, something we will cover in Section 3.3.

**Figure 4:**
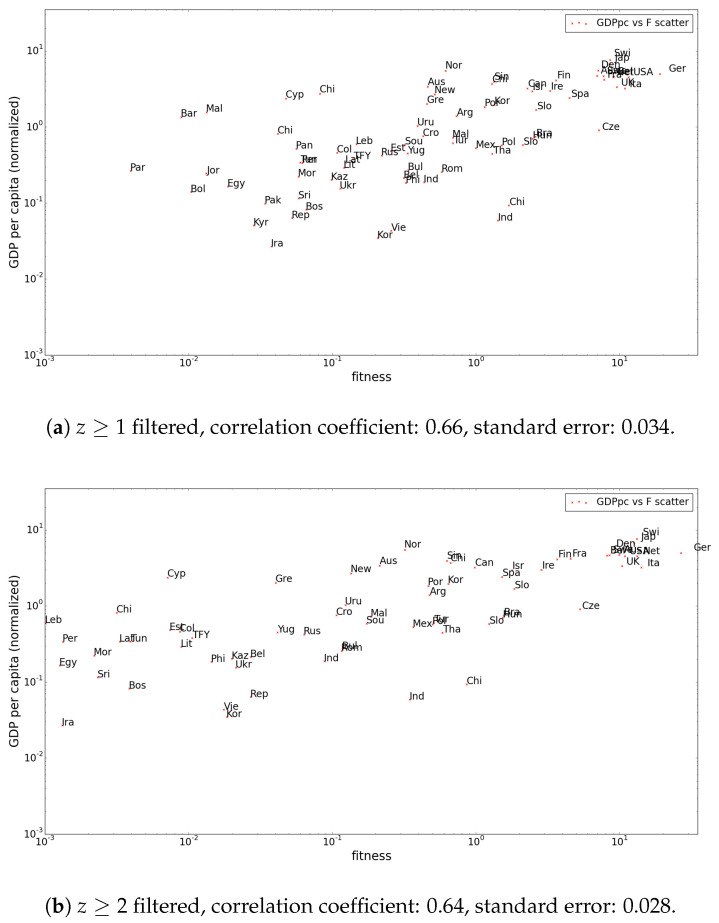
Normalized GDP per capita versus fitness (1995) plots show a similar correlation for higher *z*-score filtering thresholds. The standard error of the correlation decreases remarkably with higher thresholds (compared to 0.052 for z≥0). (**a**) z≥1 filtered, correlation coefficient: 0.66, standard error: 0.034; (**b**) z≥2 filtered, correlation coefficient: 0.64, standard error: 0.028.

These effects are also visible in the plots showing the correlation between GDP per capita and fitness of countries. Again, the top-ranked countries remain more or less stable, while countries with low initial fitness at z≥0 filtering show major variations for z≥1 and z≥2 in Figure 4. These plots point out once again the instability of the fitness and complexity algorithm as reported in [5].

A remarkable change is the much lower standard error of the correlation between GDP per capita and fitness, which can be interpreted as a justification of our approach. This also means that in an attempt to improve GDP growth forecasts using fitness, as in [7], applying the methods described in this paper could improve results.

### 3.3. Additional Results: z-Score Spectra

As a by-product of the methodology proposed in this paper, we have obtained a new type of results during our research. In a more detailed analysis of the performance of a single economy, it can be enlightening to plot the “spectrum” of the countries commodities, with their *z*-scores and complexities on the *x*- and *y*-axes, respectively.

**Figure 5:**
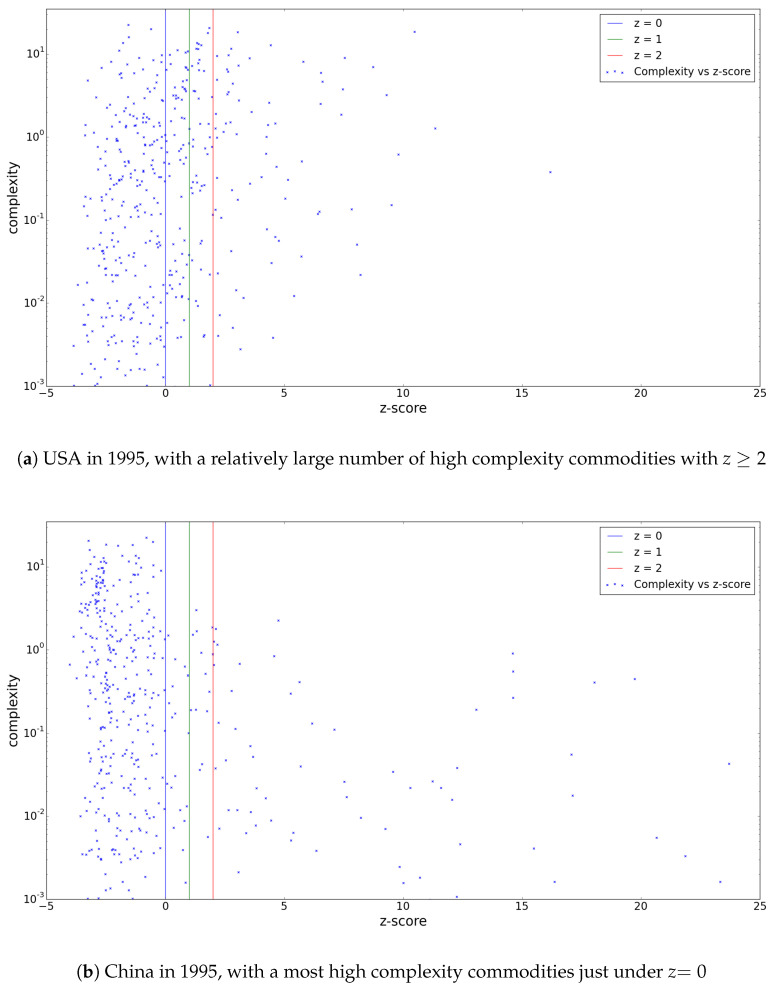
Commodity complexity spectra, showing the export product baskets of the USA and China in terms of their complexity and *z*-score. (**a**) USA in 1995, with a relatively large number of high complexity commodities with z≥2; (**b**) China in 1995, with a most high complexity commodities just under z=0.

These plots immediately yield a lot of information about the diversity of a country’s export portfolio, as can be seen in Figure 5. A diverse portfolio in this graph is, rather intuitively, represented by large numbers of complex and less complex commodities on the high *z*-score end of the spectrum, like the USA in 1995, while China in that same year shows a strongly left-leaning spectrum with only simpler products at the right end. From these spectra, it is obvious that the USA’s economy is more “robust” in the sense that it would be impacted less by a raised *z*-score threshold than China would.

One of the most interesting features of these spectra is that they can convey a country’s potential. When one finds a great number of complex commodities in a spectrum just under the usual *z*-score threshold, this could indicate that, within a reasonable time, many of these will be added to the significant export basket of this country. The plot of China’s export portfolio in Figure 5 is a good example of such a spectrum. Potentially, an analysis of the temporal evolution of such spectra could be very informative. Moreover, these spectra could point countries to what products they should invest in to improve their economical fitness (those with high complexity and a low *z*-score)—something, as was suggested by [4] that could in turn lead to an improvement in GDP.

## 4. Discussion

The above leaves a couple of items open to discuss, including a number of caveats and some potential research tangents. One is the nature of the data that we have used (for a detailed description of the data, please refer to Section 5). Firstly, the datasets only convey information on the traded commodities that an economy exports and therefore leave out all internally consumed products by definition. This is an important consideration, although one could argue that when a country really is a significant producer of a commodity, it will in most cases also export it.

A second consideration regarding the use of world trade data is that only physical products are taken into account. The whole contribution of the services sector to the global economy (around 64%) is neglected in this analysis. We will leave it to economists to estimate the importance of this neglect.

Continuing to the fitness and complexity algorithm, we have one consideration that we deem worth sharing. It could be argued that there is a slight circular reasoning to first measure the algorithm’s performance by comparison of the resulting fitness with GDP and then investigating the correlation between the both of them.

Another problem arises when choosing the configuration model to construct $p_{i,j}^{\alpha }$ with (DBCM, MDBCM or its strength-replaced counterpart). So far, we have not been able to identify a single measure to tell which model is most successful and should therefore be applied. Comparing results with GDP is not necessarily a good approach, as we are not necessarily trying to imitate it. We have opted for the (regular) MDBCM, as it was computationally faster and conceptually more appealing than the DBCM, but even more options than the three mentioned in this paper could be considered.

Lastly, one can imagine applying the fitness and complexity algorithm to importers instead of exporters, now that the new methodology offers the opportunity to construct a importer-commodity matrix as input. This was out of the scope of the current paper, but we would argue that if the complexity of the products a country exports tells us something about that country’s economy, the complexity of the products it imports would as well. Potentially, it could yield information on the wealth of a nation, so a comparison between each country’s exporting and importing fitness might prove to be an interesting next step of investigation.

## 5. Materials and Methods

### 5.1. Data

All the data that we have used are originally collected by the UN statistics division and combined in the UN Comtrade database citecomtrade. We have used the available data from the years 2004 and 2012 directly from this database [17].

We also employed parts of a longer range of data that was retrieved from Comtrade and cleaned by Robert Feenstra and Robert Lipsey from the University of California, Davis [18]. This dataset covers the years 1962 up and until 2000. This data range has been most useful for an analysis of temporal evolution of economies.

These datasets contain information of the following kind: exporter *i* trades commodity α with importer *j*, with a total value of *x* dollar. Throughout this paper, we have treated this data as a multiplex network, consisting of many individual networks per commodity combined as “layers”. These individual layers are regular weighted, directed networks. Note that, despite all efforts by Comtrade and Feenstra and Lipsey, neither of these datasets is complete, due to practical issues rather than scientific ones.

The first, more recent pair of datasets has six-digit commodity specification codes, whereas the second range of datasets consists of four-digit specifications for commodities. This means that the first database is far more detailed (including circa 5000 commodities) than the second (under 1000 commodities). This in itself has led to differences in (the nature of) our results, both within our own research and when reproducing other’s results.

This multiplex, weighted, directed network is represented by an adjacency matrix that we have called *W*. Links in this network, or rather, the value of their weights, are referred to by $w_{i,j}^{\alpha }$, with the subscripts *i* and *j* referring to the exporting and importing countries respectively, while the superscript is reserved for the layer, or commodity of the trade link.

### 5.2. Numerical Methods

All numerical efforts have been executed in Python code. For the numerical solving of the equations involving the hidden variables in $p_{i,j}^{\alpha }$, we have applied the scipy.optimize package. All other code is straightforward Python and Numpy. The code developed for our research purposes will be made publicly available to simplify reproduction of our results.

## Figures and Tables

**Table 1:** A comparison of the fitness ranking results [2,3,4] for the year 2010 and our replication for 2012. Note the differences in the bottom half of the top 10, which can be attributed to the different years that are analyzed and the different datasets.

Tacchella et al. (2010)	Replication (2012)
Germany	Germany
China	China
Italy	Italy
Japan	Japan
USA	USA
France	Belgium
UK	France
Austria	Netherlands
Spain	India
Belgium	UK

**Table 2:** Some examples of commodities with 0≤z≤1 for the Netherlands in 2004. These are allowed to pass through the original revealed comparative advantage (RCA) filter, but are denied by any filter with z≥1.

Fish (fresh or chilled)
Peas
Rubber inner tyre tubes
Sacks and bags of jute
Compacting machinery
Resistance welding machines
Electric lamps and light fittings

**Table 3:** Ranking of countries according to their fitness, in 2012.

z ≥ 0	z ≥ 1	z ≥ 2
China	China	China
Germany	Germany	Germany
USA	USA	Italy
Japan	Japan	Japan
Italy	Italy	USA
India	Belgium	Belgium
Belgium	India	India
France	France	France
Netherlands	Netherlands	Netherlands
Spain	Spain	UK
UK	UK	Spain
Hong Kong	Hong Kong	Switzerland
Switzerland	Switzerland	Hong Kong
Czech Republic	Austria	Austria
Austria	Czech Republic	Czech Republic
South Korea	South Korea	South Korea
Sweden	Sweden	Sweden
Poland	Turkey	Thailand
Turkey	Thailand	Denmark
Denmark	Malaysia	Turkey
Thailand	Denmark	Singapore
Malaysia	Poland	Malaysia

## Abbreviations

The following abbreviations are used in this manuscript:

WTN	World Trade Network
RCA	Revealed Comparative Advantage
DBCM	Directed Binary Configuration Model
MDBCM	Multiplex Directed Binary Configuration Model
